# Increased Subfoveal Choroidal Thickness and Retinal Structure Changes on Optical Coherence Tomography in Pediatric Alport Syndrome Patients

**DOI:** 10.1155/2019/6741930

**Published:** 2019-01-21

**Authors:** Seda Karaca Adıyeke, Gamze Ture, Fatma Mutlubaş, Hasan Aytoğan, Onur Vural, Neslisah Kutlu Uzakgider, Gulsah Talay Dayangaç, Ekrem Talay

**Affiliations:** ^1^Tepecik Research and Training Hospital, Ophthalmology Department, Izmir, Turkey; ^2^Tepecik Research and Training Hospital, Pediatric Nephrology Department, Izmir, Turkey

## Abstract

**Objective:**

To evaluate optical coherence tomography (OCT) findings of pediatric Alport syndrome (AS) patients with no retinal pathology on fundus examination.

**Materials and Methods:**

Twenty-one patients being followed up with the diagnosis of AS (Group 1) and 24 age- and sex-matched healthy volunteers (Group 2) were prospectively evaluated. All participants underwent standard ophthalmologic examination, retinal nerve fibre layer (RNFL) analysis, and horizontal and vertical scan macula enhanced depth imaging OCT (EDI-OCT). Statistical analysis of the data obtained in this study was performed with SPSS 15.0.

**Results:**

Macula thickness was significantly decreased in the temporal quadrant in Group 1 compared to those of the control group (*p*=0.013). RNFL measurements revealed statistically significant thinning in the temporal, superior, inferotemporal, and inferonasal quadrants and in average thicknesses in cases with AS compared to the controls (*p* < 0.001, *p* < 0.001, *p*=0.022, *p*=0.016, *p* < 0.001, respectively). The mean subfoveal coronial thickness (SCT) was 362.2 ± 77.8 *μ*m in Group 1 and 256,18 ± 71.7 *μ*m in Group 2. There was a statistically significant difference between the two groups in terms of mean CT (*p* < 0.001).

**Conclusion:**

OCT provides valuable information in identifying the structural changes and evaluation of ocular findings in patients with AS. Even if no pathological retinal findings were found in the clinical examination, structural changes in the OCT examination begin in early period of AS.

## 1. Introduction

Alport syndrome (AS) is a hereditary disease resulting from basal membrane type 4 collagen disorder, characterized by progressive renal dysfunction, sensorineural hearing loss, and ocular anomalies [1, 2]. In the majority of families with AS (85%), there are mutations in the X-linked COL4A5 gene. Mutations of the COL4A4 and COL4A3 genes cause autosomal recessive and, rarely, autosomal dominant AS [3, 4]. A characteristic histopathological feature is progressive degeneration of the basal membranes in the eyes, ears, and kidneys [3].

In AS, fleck retinopathy and macular thinning, especially affecting the inner retinal layers, occur due to type 4 collagen impairment [5]. Retinal flecks may involve the central or peripheral retina. The centrally located flecks may appear in the form of a perifoveal ring and may cause a glare. This finding is called “Lozenge.” In AS, the Lozenge finding has been shown to be associated with early renal insufficiency [6].

The most commonly reported eye findings in AS are anterior lenticonus, fleck retinopathy, cataracts, and posterior polymorphous corneal dystrophy [7]. Apart from these findings, recurrent corneal epithelial erosion, spontaneous lens anterior capsular rupture, posterior lenticonus, a giant macular hole, and temporal macular thinning have been reported in AS [8, 9].

Ocular disorders of AS rarely occur in childhood. Due to the progressive nature of the disease, the ocular findings of AS are more prevalent in adult patients [10].

The aim of our study was to evaluate optical coherence tomography (OCT) findings of pediatric AS patients with no retinal pathology on a fundus examination.

## 2. Materials and Methods

### 2.1. Study Design and Population

Twenty-one patients aged 9 to 18 years (Group 1) who were followed up with in the Department of Pediatric Nephrology with a diagnosis of AS were included in the study. The patients were evaluated prospectively in this study between October 2016 and April 2017. In total, 24 healthy age- and sex-matched volunteers (Group 2) constituted the control group.

The study was carried out in accordance with the Helsinki Criteria and approved by the Local Ethics Committee. All participants or their legal representatives have signed the informed consent form.

All the cases in Group 1 underwent mutation analyses. Detailed medical histories of all cases were assessed. Demographic data, including age, past medical history, sex, smoking status, and systemic and/or topical drug use, were recorded. A standard ocular examination, macula-enhanced depth imaging (EDI)-OCT, and retinal nerve fiber layer (RNFL) analyses were performed on all of the subjects. At baseline, all patients underwent a best-corrected visual acuity (BCVA) measurement by means of Snellen charts. BCVA measurements were converted to logarithm of the minimum angle resolution (LogMAR) visual acuity (VA) for analysis.

The Alport patients with renal insufficiency, retinal flecks, and the lozenge sign were excluded from the study. Patients with systemic diseases other than AS, with abnormal hormone levels, and who did not undergo genetic analysis were excluded from the study.

In all cases, an OCT examination was performed by the same staff with a spectral domain (SD)-OCT device (Spectralis OCT, Heidelberg Engineering, Heidelberg, Germany) in our clinic. SD-OCT in enhanced depth imaging mode was used for all OCT scans.

### 2.2. Macula Thickness Measurement and RNFL Analyzes

The macula was screened by obtaining 49 sections (512 A-scan). An average of nine images was obtained for each section. The fovea was evaluated using vertical and horizontal scans with an average of 100 images.

The macular thickness was evaluated automatically and measured using the Spectralis software in the nasal, inferior, temporal, and superior quadrants of the 1 mm circle around the fovea, as well as in the 1–3 mm-wide annular area located pericentrally.

In the OCT examination, the internal limiting membrane (ILM), RNFL, and retina pigment epithelium (RPE) hyper-reflectivity, and RPE/Bruch's membrane complex irregularity were evaluated in vertical and horizontal scans.

In the OCT examination, the RNFL thicknesses were assessed by scanning a peripapillary circle with a diameter of 3.4 mm and 768 A-scans. The RNFL thicknesses were automatically segmented and were measured using the Spectralis software.

### 2.3. Subfoveal Choroid Thickness Measurement

Choroidal imaging was performed as the vertical and horizontal scans. The SCT was defined as the vertical perpendicular distance from the hyper-reflective line of Bruch's membrane to the innermost hyper-reflective line of the choroidal-scleral interface (CSI). The measurements were taken at the fovea, 500 *µ*m nasal and temporal to the fovea in horizontal scans, and 500 *µ*m superior and inferior to the fovea in vertical scans. The mean SCT was calculated by taking the average of these five values. The images were obtained with the best visualization of the border between the choroid and the sclera, known as the CSI. To avoid possible diurnal variation in choroidal thickness, all EDI-OCT examinations were performed between 9 AM and 12 AM.

We measured intraobserver repeatability using two OCT images obtained by the same operator; interobserver reproducibility was measured using two OCT images obtained by two different operators. We calculated the overall mean thickness and intraclass correlation coefficients (ICCs) to evaluate the repeatability and reproducibility of the thickness measurements. ICC estimates and their 95% confidence intervals were calculated based on a mean rating (*k*=2), absolute-agreement, two-way random-effects model. ICC values greater than 0.90 were considered to show excellent reliability.

## 3. Statistical Analysis

For statistical analysis, this study used the Statistical Package for the Social Sciences (version 17.0; SPSS, Chicago, IL). To summarize the study data, we used descriptive statistics (e.g., mean, standard deviation, range, frequency, and percentage). We used the Kolmogorov–Smirnov and Shapiro–Wilk tests to evaluate the distribution of participants in Groups 1 and 2. For a comparison of groups, we used the independent *t*-test and Mann–Whitney test for variables. The limit for statistical significance was *p* < 0.05.

## 4. Results

Twenty-one subjects diagnosed with AS (Group 1) and 24 healthy controls (Group 2) were included in the study. Group 1 consisted of 12 females and 9 males, while 11 females and 13 males formed Group 2.

A single mutation in the COL4A4 gene was detected in three male and 10 female patients, a single COL4A3 gene mutation was detected in two male and two female patients, and X-linked Alport syndrome (COL4A5 gene mutation) was detected in four male patients.

One of the 21 patients was using Angiotensin converting enzyme inhibitors in Group 1 due to continued proteinuria, while the rest did not receive any medication. None of our cases developed end-stage renal failure. None of the participants was smokers.

The mean BCVA in the Alport group was 0.012 ± 0.038 logMAR (range: 0.15–0.0), while BCVA was 0.0 logMAR in all of the control subjects. The difference between the two groups with respect to BCVA was statistically insignificant (*p*=0.07).

The mean age of Group 1 was 14.6 ± 2.51 years (range: 9–18 years) and that of Group 2 was 13.8 ± 3.37 years (range: 10–18 years). The study groups were comparable in terms of age.

Posterior polymorphous corneal dystrophy, recurrent corneal epithelial erosion, posterior lenticonus, a giant macular hole, or conjunctival telangiectasia was not detected in any of our cases.

Two siblings in Group 1 had anterior lenticonus, and the anterior segment OCT revealed a bilateral anterior capsular rupture in one of the siblings.

The lozenge finding was not detected in any cases.

**Figure 1:**
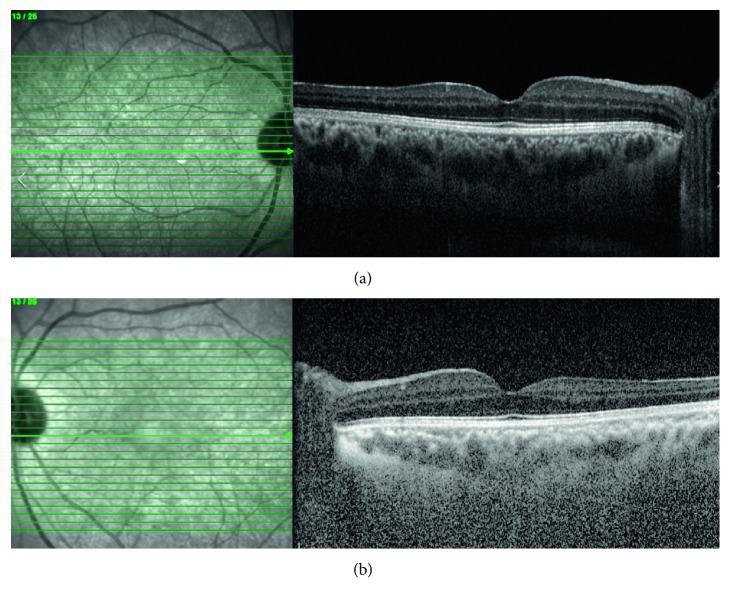
EDI-OCT scan of a 20-year-old male patient. Temporal foveal thinning is seen in right eye (a) and left eye (b) of the patient.

In the horizontal scan macular OCT examinations, morphological thinning was observed in the temporal half of the fovea in two cases (Figure 1). When macular OCT thickness measurements are evaluated, in the 1–3 mm-wide annular area located pericentrally, the macula thickness was significantly decreased in the temporal quadrant in the Alport group compared to the control group (*p*=0.003). There was no significant difference between the groups in the other quadrants. There was no statistically significant difference between the two groups in terms of the macular thickness in the fovea-centered circle, which was 1 mm in diameter. The data regarding macular thicknesses are summarized in Table 1.

**Figure 2:**
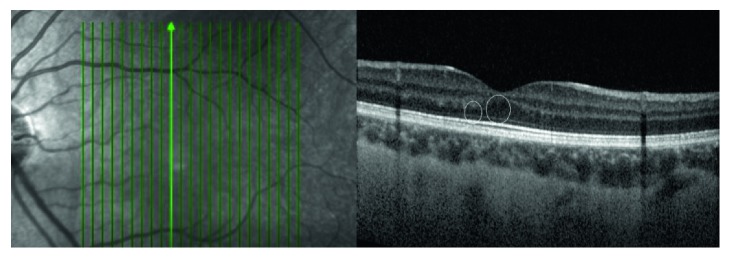
Very small cyst-like occurrences are observed in the outer nuclear layer of a 15-year-old case.

**Figure 3:**
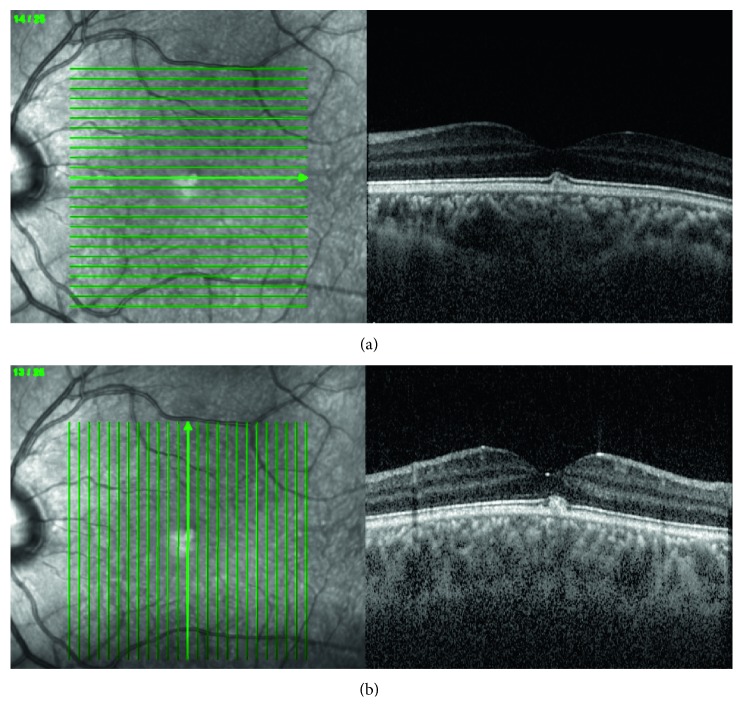
Temporal and horizontal OCT scan of a 16-year-old patient. Subfoveal vitelliform lesion is observed in horizontal (a) and vertical scans (b).

RNFL, RPE, and ILM hyper-reflectivity were not detected in the cases. Although not detected in the retina examination, in one case, small cystic cavities were observed in the outer nuclear layer (Figure 2). In another case, a vitelliform deposit was seen in one eye on fundus examination and on macular OCT scans (Figure 3). In these two cases, VA was measured as 0.0 logMar. No morphological changes were detected on the OCT examination, and all retinal layers were observed as normal in the other 17 cases.

Demographic and clinical findings in patients with AS are summarized in Table 2.

Vitreous degeneration or vitreoretinal interface abnormalities were not detected in any of our cases. In the horizontal macular OCT scans, RPE/Bruch's membrane complex and cost line were regular, and no pathology was detected.

The mean subfoveal CT value was measured as 362.2 ± 77.8 *μ*m (range 256–473 *μ*m) in Group 1 and 256.18 ± 71.7 *μ*m (range 234–385 *μ*m) in Group 2. The subfoveal CT of the AS group was significantly higher than that of the control group (*p* < 0.001).

Hyper-reflective dots were not found in any of the cases.

RNFL measurements revealed statistically significant thinning in the temporal, superotemporal, inferotemporal, and inferonasal quadrants and average thicknesses in cases with AS compared to the controls (*p* < 0.001, *p* < 0.001, *p*=0.022, *p*=0.016, *p* < 0.001, respectively). There was no significant difference between the two groups in the other quadrants. The RNFL thickness values found in all quadrants are summarized in Table 3.

## 5. Discussion

A defect in the synthesis of type IV collagen leads to abnormal basement membrane formation in AS. Type IV collagen is also present in the basement membranes of the ILM, Bruch's membrane, the RPE, and the choriocapillaris layer.

The most frequent findings are anterior lenticonus and flecking related to lozenge findings. Posterior polymorph corneal dystrophy, recurrent epithelial lesions, spontaneous anterior lens capsule rupture, a giant macular hole, and temporal thinning are other less common findings [11, 12]. Flecked retinopathy and thinning in the temporal macula has not been shown to cause any deterioration in visual functions [12]. Lozenge findings are important in the diagnosis of AS, and it has been determined that its prognostic value is important in the detection of early-onset renal failure [11].

Ocular findings of AS usually occur after the adolescence period [10]. In our study, although no fundus pathology was detected in the biomicroscopic ocular examination, OCT revealed pathologic findings in pediatric AS patients. In addition to the temporal macula thinning and vitelliform lesions previously described in the literature, we showed that choroidal thickness was increased in these patients.

In our study group, we detected a COL4A5 gene mutation in four patients, all of whom were boys. Because the majority of our group was composed of girls, we had seen mostly COL4A4 and COL4A3 gene mutations, which reflected the autosomal recessive inheritance.

The value of the vertical macular OCT scans increases in the diagnostic work-up for retinal diseases. The vertical macular OCT scan is used to evaluate abnormalities, including the macular hole configuration, [13] posterior precortical vitreous structure and dissociation pattern, [14] and optical nerve head anomalies [15]. In addition, a vertical OCT scan is important in the diagnosis of dome-shaped staphyloma in high myopia, peripapillary intracoroidal cavitation, and retinoschisis [16, 17]. Dotz-Marco et al. assessed two cases with AS at an adult age. They reported temporal macular thinning in the horizontal macular OCT scans of these cases, whereas their vertical OCT scans did not detect any pathology [18]. For this reason, we performed vertical and horizontal macular OCT scans of our cases. In two cases, we observed temporal thinning only on horizontal OCT scans.

Thinning in the temporal macula in AS was reported in a case by Usui et al. and in a 10-case series by Savig et al. previously [5, 19]. Usui et al. reported that the visual field test and multifocal electroretinography (ERG) examination were normal in this patient, who revealed temporal thinning and whose visual function was not affected. In their study, Savig et al. found that there was an increase in reflectivity in the ILM and RNFL layers in accordance with the distribution of flecking retinopathy [5]. Researchers have shown that the ILM and Bruch's membrane thicknesses were reduced. ILM and RNFL thinning are thought important in the development of retinopathy, as the ILM is a barrier to the vitreous traction forces and plays a role in waste and nutrient transfer.

Similar to the literature, [19] we observed that the temporal macular thickness was decreased without a significant effect on VA in our cases. Except for two siblings with anterior lenticonus, the BCVA values of all participants were 0.0 logMar.

In our study, we observed that the choroidal thickness was increased in patients with AS compared to the control group. An increased choroidal thickness in our AS cases is thought to be related to over-expressed proinflammatory cytokines (interleukin 6, tumor necrosis factor *α*, and interleukin 1−*β*). The increase in inflammatory cytokines through various cascades is defined in AS [20, 21].

It has been reported that vitelliform lesions, small cystic cavities, and serous retinal detachment are observed, in addition to retinal thinning and macular holes [22–25].

The genetically altered collagen component of the basement membrane of the retinal pigment epithelium may be concerned with the pumping function of retinal pigment epithelium (RPE) cells and could thus play a role in the genesis of serous retinal detachments, vitelliform lesions, small cystic cavities.

It has been shown that abnormal fluid passage due to Bruch's membrane and RPE dysfunction plays a role in the pathophysiology of full-thickness macular holes in small cystic cavities formed in the retinal layers [23]. It was reported that these findings might be due to the thinning and dissection of the basal membrane due to loss of type 4 collagen, impairments in Bruch's membrane, the ILM and vitreous interface, and abnormal vitreous traction [5, 22].

The accumulation of these fluid vacuoles can lead to serous retinal detachment [26]. Hypertensive retinochoroidopathy, hypoalbuminemia, and dilutional hyponatremia have been shown to play an important role in serous retinal detachment development [27, 28].

Vitelliform lesions have also been reported to result from the accumulation of side products of photoreceptor outer segments. Fawzi et al. reported vitelliform lesions in two of nine adult cases [29]. These lesions were thought to be due to the accumulation of lipofuscin and melanolipofuscin resulting from a defective Bruch's membrane with a similar pathophysiological process seen in age-related macular degeneration [29].

Vitelliform lesions, small cystic cavities, and temporal thinning on an OCT examination have been detected only in adult patients in previous studies. In our study, we determined that these changes were observed in pediatric cases and were seen without renal insufficiency. Cystic cavities and temporal retinal thinning have not any effect on visual acuity, but these can be pioneering lesion of giant macula hole. Vitelliform lesions and cystic cavities are also an indicator of RPE pumping disfunction and can develop serous retinal detachment if hypertensive retinochoroidopathy, hypoalbuminemia, and dilutional hyponatremia are present. Therefore, regular ophthalmologic and OCT monitoring of pediatric cases with AS are important. Case follow-up should be done in coordination with nephrology, and the initiation of pathological findings in OCT can be considered in nephrological follow-up and treatment.

In our cases, we did not observe vitreous degeneration or vitreous interface abnormalities. A small number of cases and a lack of electrophysiological tests are the limitations of our study. Further large-scale studies are needed to reveal the relationship between genetic mutation types and OCT findings.

OCT provides valuable information for identifying structural changes and evaluating ocular findings in patients with AS. The structure changes of the retinal and choroidal layers begin in the early period of AS. Therefore, it is important to evaluate and follow up on these cases using an OCT examination, even if no pathological retinal findings were revealed in the clinical examination.

## Figures and Tables

**Table 1:** Macular thickness values for each group at the 1 mm diameter circle centered on the fovea and the parafovea 1–3 mm diameter circle in four quadrants.

	Alport syndrome group (*n*=21) (mean ± SD) (*μ*m)	Control group (*n*=24) (mean ± SD) (*μ*m)	*p* value
TIM	289.40 ± 21.80	297.19 ± 26.44	0.013^*∗∗*^
SIM	291.04 ± 23.30	295.18 ± 33.93	0.067
NIM	293.07 ± 21.08	297.46 ± 21.47	0.07
IIM	288.83 ± 22.80	290.14 ± 20.78	0.130
CST	238.46 ± 30.1	241.68 ± 31.2	0.562

TIM: temporal inner macula, SIM: superior inner macula, NIM: nasal inner macula, IIM: inferior inner macula, and CST: central subfield thickness.. ^*∗∗*^Statistically significant *p* value.

**Table 2:** Demographic and clinical findings in patients with Alport syndrome.

No	Age	Sex	Genetic mutation	Clinical and OCT findings	Sph equ RE	Sph equ LE
1	17	M	COL4A4	Unilateral vitelliform lesion	+0.75	−0.50
2	18	F	COL4A4	None	−0.25	0.00
3	14	F	COL4A4	None	+0.25	+0.25
4	11	M	COL4A3	None	+0.50	+0.375
5	18	M	COL4A5	Temporal macula thinning	−0.75	−1.00
6	16	M	COL4A5	Temporal macula thinning	+0.25	−0.75
7	15	F	COL4A4	None	+0.50	+1.00
8	18	M	COL4A5	None	−0.25	0.00
9	9	M	COL4A5	None	−0.375	−0.50
10	12	F	COL4A4	None	+0.25	+0.125
11	15	M	COL4A4	None	−0.50	−0.25
12	12	F	COL4A4	None	+0.75	+0.75
13	17	F	COL4A3	None	0	−0.375
14	13	M	COL4A4	None	+0.875	+0.625
15	13	F	COL4A4	None	+0.25	+0.25
16	12	F	COL4A4	None	+0.25	0
17	15	F	COL4A4	None	+1.25	+1.00
18	14	F	COL4A3	None	+0.50	+0.25
19	16	F	COL4A4	None	+0.125	+0.375
20	15	F	COL4A4	None	+0.75	+0.50
21	17	M	COL4A3	None	−0.375	−0.875

M: male, F: female, Sph equ: spherical equivalent (diopter), RE: right eye, LE: left eye.

**Table 3:** RNFL thickness measurements in Alport syndrome and control groups.

	Alport syndrome group (*n*=21) (mean ± SD) (*μ*m)	Control group (*n*=24) (mean ± SD) (*μ*m)	*p* value
RNFLt	65.92 ± 11.78	73.58 ± 8.20	0.001
RNFLs	127.38 ± 19.38	144.02 ± 14.57	0.001
RNFLsn	118.52 ± 14.63	121.28 ± 22.39	0.811
RNFLn	82.23 ± 9.71	80.06 ± 13.18	0.270
RNFLin	114.50 ± 20.58	129.6 ± 20.6	0.016
RNFLit	131.09 ± 16.02	153.48 ± 18.96	0.022
RNFLav	99.95 ± 7.75	106.62 ± 8.42	0.001

## Data Availability

The data used to support the findings of this study are available from the corresponding author upon request.

## Additional Points

*Brief Summary Statement*. It is important to evaluate the choroidal thickness and the retinal structure morphology carefully on the OCT examination of pediatric Alport syndrome patient even if no pathological retinal findings were found in the clinical examination.
